# Sex- and region-specific associations of skeletal muscle mass with metabolic dysfunction-associated fatty liver disease

**DOI:** 10.3389/fendo.2022.1057261

**Published:** 2022-12-02

**Authors:** Pei Xiao, Pu Liang, Panjun Gao, Jinyi Wu

**Affiliations:** ^1^ Center for Non-Communicable Disease Management, Beijing Children’s Hospital, Capital Medical University, National Center for Children’s Health, Beijing, China; ^2^ Beijing Key Laboratory of Emerging Infectious Diseases, Institute of Infectious Diseases, Beijing Ditan Hospital, Capital Medical University, Beijing, China; ^3^ Beijing Institute of Infectious Diseases, Beijing, China; ^4^ National Center for Infectious Diseases, Beijing Ditan Hospital, Capital Medical University, Beijing, China; ^5^ Department of Health, Ethics & Society, CAPHRI Care and Public Health Research Institute, Faculty of Health, Medicine and Life Sciences, Maastricht University, Maastricht, Netherlands; ^6^ Department of Public Health, Wuhan Fourth Hospital, Wuhan, China

**Keywords:** metabolic dysfunction-associated fatty liver disease, fibrosis, skeletal muscle mass, body composition, National Health and Nutrition Examination Survey

## Abstract

**Introduction:**

Metabolic dysfunction-associated fatty liver disease (MAFLD) is known to be the most common chronic liver disease worldwide, and accumulating evidence suggests that skeletal muscle might play an important role in metabolic health. However, the association between skeletal muscle and MAFLD is poorly understood so far. Therefore, we aimed to evaluate the associations of skeletal muscle with MAFLD and significant fibrosis.

**Methods:**

A cross-sectional analysis was conducted using data obtained from the 2017-2018 US National Health and Nutrition Examination Survey. The whole-body, appendicular, and trunk skeletal muscle mass index (SMI) were assessed by dual-energy x-ray absorptiometry. MAFLD and significant fibrosis were assessed by transient elastography. Survey-weight adjusted multivariable logistic regressions were used to determine the associations. The area under the receiver operating characteristic curve (AUC) and variable importance scores from the random forest and logistic regression model were calculated to assess the predictive capability of variables and models.

**Results:**

Of the 2065 participants, those with appendicular SMI in the highest quartile were associated with a lower risk for MAFLD in both sexes (male, OR[95%CI]: 0.46 [0.25~0.84]; female, OR[95%CI]: 0.32 [0.13~0.82]), but with a significantly different scale of the associations between sexes (*P*_interaction_ = 0.037). However, females with trunk SMI in the highest quartile had an increased risk of significant fibrosis (OR[95%CI]: 7.82 [1.86~32.77]). Trunk SMI and appendicular SMI ranked the third contributor to MAFLD in random forest and logistic regression models, respectively. Taking appendicular and trunk SMI into consideration, the AUCs for MAFLD were 0.890 and 0.866 in random forest and logistic regression models, respectively.

**Discussion:**

The distribution of skeletal muscle mass differently affects MAFLD and significant fibrosis in the sex groups. Higher appendicular skeletal muscle mass was associated with a lower risk of MAFLD, while the risk of significant fibrosis in females was increased with the trunk skeletal muscle mass.

## Introduction

Metabolic dysfunction-associated fatty liver disease (MAFLD), formerly named non-alcoholic fatty liver disease (NAFLD), is the most common cause of chronic liver disease affecting approximately one-quarter of the adult population worldwide (1). Given the high correlation between fatty liver and metabolic disease, the newly named MAFLD is proposed to be diagnosed by the presence of hepatic steatosis and coexistence with any of the following three conditions: overweight/obesity, metabolic risk abnormalities, or type 2 diabetes mellitus (T2DM). Over the decades, it causes an enormous and increasing burden on global health, and yet there is no approved pharmacologic treatment. Around 2 out of 5 adults in the US are suffering from MAFLD, and they have a significantly higher risk of all-cause and cardiovascular mortality (2). Moreover, the rising trend of obesity and T2DM will fuel a growing epidemic of MAFLD globally (3). Therefore, further understanding of emerging risk factors contributing to MAFLD might help to identify individuals at higher risk and develop prevention or intervention strategies.

Different body weight compartments may play opposite roles in the progress of disease: fat mass is detrimental to health, whereas skeletal muscle mass has the disposition to promote healthy outcomes (4). A large cohort of UK adults has revealed that greater muscle quality was associated with decreased risk of cardiovascular mortality (5). In addition, muscle weakness has been observed to be associated with T2DM, which was an important component of MAFLD (6). As an endocrine organ, skeletal muscle can secrete myokines that might affect the function of the liver (7). Nevertheless, the relationships between skeletal muscle mass and NAFLD were estimated only by a limited number of studies and using less accurate surrogate measures, such as handgrip strength and hepatic steatosis index (HSI) (8, 9). Moreover, the effects of regional skeletal muscle distribution on MAFLD and the differences between sexes are largely unknown.

Considering the significant impacts of MAFLD and the knowledge gap between MAFLD and skeletal muscle mass, a better understanding on the characteristics of skeletal muscle mass in individuals with MAFLD could help to identify the susceptible population and control the progression of this metabolic disease. Using national representative data of body composition, and hepatic steatosis and fibrosis in the US, we therefore aimed to determine whether skeletal muscle mass and its distribution were associated with the decreased risk of MAFLD and significant fibrosis, and to investigate the prognostic impact of genders on MAFLD progression.

## Material and methods

### Study population

**Figure 1:**
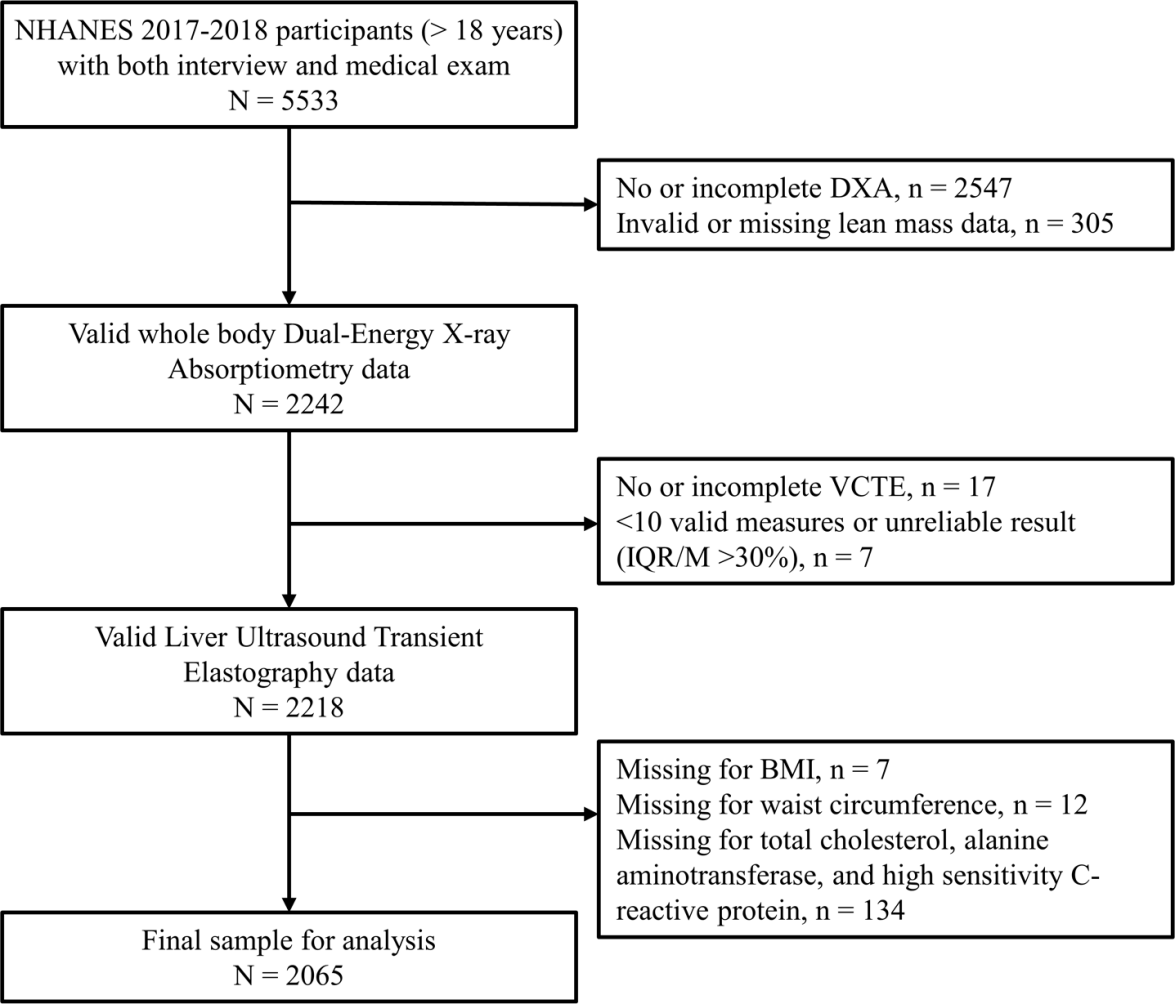
Flow diagram of the analytic sample.

The data was obtained from the recent 2017-2018 cycle of the National Health and Nutrition Examination Survey (NHANES). It is a program of studies to assess the health and nutritional status of adults and children in the US using a multi-stage, stratified, and clustering probability sampling method (10). A total of 9254 participants were included in the NHANES 2017-2018 public release data. Among them, 5533 adults (> 18 years) have been both interviewed and examined at a mobile center. After excluding individuals (n = 3468) with missing or invalid data for dual-energy x-ray absorptiometry (DXA), vibration controlled transient elastography (VCTE), body measures, total cholesterol, and alanine aminotransferase, 2065 participants with complete data were included in the final analysis (**Figure 1**). All participants provided informed consent, and the protocols of NHANES were approved by the Centers for Disease Control and Prevention institutional review board. The current study was exempted by the institutional review board due to the use of entirely de-identified data.

### Data collection

Detailed information about the 2017-2018 cycle of the NHANES protocol can be found online (https://www.cdc.gov/nchs/nhanes/index.htm). Briefly, a combination of computer-assisted personal interviewing and audio computer-assisted self-interviewing system were used to obtain sociodemographic and behavioral characteristics, including race, education, poverty, smoking, drinking, physical activity, eating habits, and history of cardiovascular disease. A standardized health examination, which included physical examination and laboratory tests, was conducted at a mobile examination center.

### MAFLD and significant fibrosis

Hepatic steatosis and liver fibrosis, as two important liver disease manifestations, were objectively assessed by the liver ultrasound transient elastography. The controlled attenuation parameter (CAP) related to the presence of hepatic steatosis and liver stiffness measurement (LSM) related to liver fibrosis were recorded by FibroScan^®^ (Echosens, Waltham, MA) which used ultrasound and the VCTE (11). Participants were considered to be eligible for the examination if they fasted at least 3 hours prior to the exam and had 10 or more complete stiffness measures with a liver stiffness interquartile (IQR) range/median < 30%. The inter-observer reliability was 0.94 for the CAP score and 0.86 for LSM. We define MAFLD as the presence of hepatic steatosis (CAP scores ≥ 263 dB/m) (12) with at least one of the following: (1) overweight or obesity (body mass index(BMI) ≥ 25 kg/m^2^), (2) T2DM, or (3) at least 2 metabolic risk abnormalities (1). Metabolic risk abnormalities consisted of (1) waist circumference ≥ 102 cm for men or ≥ 88 cm for women, (2) blood pressure ≥ 130/85 mmHg or specific drug treatment, (3) fasting plasma triglycerides (TG) ≥ 150 mg/dl or specific drug treatment, (4) plasma high density lipoprotein cholesterol (HDL-C) <40 mg/dl for men or <50 mg/dl for women or specific drug treatment, (5) prediabetes, for example, fasting glucose levels between 100 and 125 mg/dl, or hemoglobin A1c between 5.7% and 6.4%, (6) homeostasis model assessment of insulin resistance score ≥ 2.5, (7) plasma high-sensitivity C-reactive protein level >2 mg/L. Significant fibrosis was defined as a median LSM ≥ 8 kPa (13, 14). Besides, we also defined the hepatic steatosis of MALFD as CAP scores ≥ 274 dB/m to perform a sensitivity analysis (14).

### Skeletal muscle mass and its distribution

The data on body composition was collected by DXA scans (Hologic, Inc., Bedford, Massachusetts) with the Hologic software of APEX v4.0. DXA is the most widely accepted method due to its speed, ease of use, and low radiation exposure. Data was considered invalid if the participants were removable, non-removable, or morbidly obese. Skeletal muscle mass referred to the lean mass excluding bone mineral content recorded by DXA. Skeletal muscle mass index (SMI) was defined as skeletal muscle mass by height squared (kg/m^2^). Appendicular SMI was defined as the sum of both arms and legs skeletal muscle mass divided by height squared (kg/m^2^). Trunk SMI was defined as trunk skeletal muscle mass by height squared (kg/m^2^). In the current study, we defined adequate skeletal muscle mass status as the SMI in the highest quartile.

### Covariates

The race was defined as non-Hispanic White, non-Hispanic Black, Hispanic, or other. Educational status was categorized into < 12th grade, high school graduate, and college or above. Poverty was defined as the family income-to-poverty ratio ≤ 0.99. Marital status was grouped as being married or living with a partner vs others. Smoking behaviors were grouped as current smoker, former smoker, and nonsmoker. Among participants who had smoked at least 100 cigarettes in their lifetime, current smoker was defined as those who reported still smoking every day or some days at the time of survey, whereas former smoker referred to those who did not smoke at the time of survey (15). Alcohol consumption was assessed by asking how many days of the year they drank alcoholic beverages and the average number of drinks consumed on drinking days during the past year. We categorized alcohol consumption into three groups: no past year use, light-to-moderate (≤ 2 drinks/day for men or ≤ 1 drink/day for women), and heavy use (16). We calculated the Health Eating Index (HEI)-2015 by using the US Department of Agriculture Food Patterns Equivalents Database and 24-h dietary recalls collected in NHANES (17). With a total score ranging from 0 to 100, the HEI-2015 assesses the adequacy of the consumption of foods as a healthy diet, which consists of 13 components: 9 adequacy components (total fruits, whole fruits, total vegetables, greens and beans, whole grains, dairy, total protein foods, seafood and plant proteins, and fatty acids) and 4 moderation components (refined grains, sodium, added sugars, and saturated fats). Global Physical Activity Questionnaire was used in the survey to assess the typical physical activity over the past week. We calculated the sum of metabolic equivalent (MET) hours per week across all physical activity using suggested MET scores in NHANES (18). Weight status was categorized into underweight or normal (BMI < 25 kg/m^2^), overweight (BMI 25-29.9 kg/m^2^), and obesity (BMI ≥ 30 kg/m^2^). Hypertension was defined as systolic blood pressure ≥ 130 mmHg/diastolic blood pressure ≥ 85 mmHg, or currently using specific drugs. T2DM was diagnosed by fasting glucose > 125 mg/dl, hemoglobin A1c ≥ 6.5%, or self-reporting. Fat mass index (FMI) was defined as fat mass by height squared (kg/m^2^).

### Statistical analysis

Given a complex, multi-stage sample design used in the NHANES, we applied appropriate sample weights in all analyses to account for clustering, stratification, non-response, and oversampling population. The continuous variables were described as the weighted mean ± SE, and the categorical variables were presented as the number (weighted frequency). Rao-Scott χ^2^ test and *t*-test were used to compare the differences in categorical and continuous variables between groups, respectively. We applied sex-specific generalized additive models with smoothing spline terms to explore the nonlinear associations of SMI, appendicular SMI, and trunk SMI with CAP scores and LSM. Restricted cubic spline regression models with three knots were used to further examine the nonlinear relationships between muscle mass and MAFLD, as well as significant fibrosis. Survey-weight adjusted multivariable logistic regressions were performed to determine the independent associations between the adequate muscle mass status (dichotomous variable) and MAFLD or significant fibrosis. We further added a two-way product term of adequate muscle mass status and sex into the models to evaluate the interactions between them. In the multivariate analyses mentioned above, sex (except for sex-stratified analysis), age, race, education, poverty, married status, smoking, alcohol use, HEI, physical activity, BMI, hypertension, waist circumference, total cholesterol, alanine aminotransferase (ALT), and FMI were treated as covariates. To further determine the impact of these factors on the MAFLD and significant fibrosis, we calculated the area under the receiver operating characteristic curve (AUC) and variable importance scores from the random forest and logistic regression model.

A 2-tailed *P* ≤0.05 was considered statistically significant, and the analyses were performed using the “survey” and “caret” package of R software (R Foundation for Statistical Computing, Vienna, Austria) with Taylor series linearization.

## Results

### Characteristics of study population

A total of 2065 participants were included in the analysis. The weighted prevalence of MAFLD and significant fibrosis were 39.2% (95%CI 36.4%-42.0%) and 6.1% (95%CI 4.8%-8.0%), respectively. The characteristics of study population by MAFLD and MAFLD with fibrosis are presented in **Table 1**. Compared to those without MAFLD, MAFLD subjects were older and had significantly higher levels of SMI, total cholesterol, and LDL cholesterol. In addition, they had lower health eating index and physical activity (*P <*0.05). Among the MAFLD patients, individuals with significant fibrosis had significantly higher levels of SMI, appendicular SMI, and trunk SMI (*P <*0.05).

**Table 1:** Characteristics of study sample.

Characteristics	All	MAFLD			MAFLD with significant fibrosis			
		No	Yes	*P* value	No		Yes	*P* value
No, %	2065 (100.0)	1204 (60.8)	861 (39.2)		761 (88.4)		100 (11.6)	
Male ^a^	1019 (51.4)	531 (47.2)	488 (58.0)	0.004	426 (56.0)		62 (62.0)	0.301
Age, y ^b^	37.6 (12.3)	35.4 (12.2)	41.0 (11.7)	<0.001	40.7 (11.9)		44.7 (11.1)	0.001
Race/ethnicity ^a^								
Non-Hispanic White	536 (20.4)	383 (59.8)	239 (52.2)	0.006	240 (31.5)	31 (31.0)		0.922
Non-Hispanic Black	622 (56.8)	250 (11.1)	143 (9.3)		212 (27.9)	27 (27.0)		
Hispanic	393 (10.4)	265 (17.1)	271 (25.5)		124 (16.3)	19 (19.0)		
Other	514 (12.4)	306 (12.0)	208 (12.9)		185 (24.3)	23 (23.0)		
Education ^a^								
<12th grade	336 (10.1)	179 (9.2)	157 (11.5)	0.243	135 (17.7)	22 (22.0)		0.382
High school graduate	567 (28.0)	354 (28.9)	213 (26.7)		193 (25.4)	20 (20.0)		
College or above	1162 (61.9)	671 (61.9)	491 (61.8)		433 (56.9)	58 (58.0)		
Poverty ^a^	389 (13.0)	252 (14.4)	137 (10.7)	0.101	120 (15.8)	17 (17.0)		0.864
Married status ^a^	1299 (61.4)	728 (57.1)	571 (68.0)	<0.001	500 (65.7)	71 (71.0)		0.347
Smoking ^a^								
Current Smoker	386 (18.2)	236 (19.0)	150 (17.0)	0.319	130 (17.1)	20 (20.0)		0.761
Former Smoker	305 (19.5)	149 (17.8)	156 (22.2)		139 (18.3)	17 (17.0)		
Nonsmoker	1374 (62.3)	819 (63.2)	555 (60.8)		492 (64.7)	63 (63.0)		
Alcohol use ^a^								
No past year use	562 (21.3)	324 (19.6)	238 (24.0)	0.106	209 (27.5)	29 (29.0)		0.536
Light-to-moderate	673 (34.5)	377 (33.3)	296 (36.3)		258 (33.9)	38 (38.0)		
Heavy use	830 (44.2)	503 (47.1)	327 (39.7)		294 (38.6)	33 (33.0)		
Healthy eating index ^b^	48.8 (13.5)	49.7 (13.8)	47.3 (12.8)	0.032	48.2 (12.9)	49.4 (14.0)		0.38
Physical activity, MET hours/week ^b^	94.1 (126.9)	98.9 (132.5)	86.6 (117.5)	0.066	92.6 (128.1)	66.5 (89.1)		0.048
Weight status ^a^								
Normal or underweight	699 (33.2)	647 (51.5)	52 (4.8)	<0.001	48 (6.3)	4 (4.0)		<0.001
Overweight	675 (32.9)	375 (33.0)	300 (32.6)		283 (37.2)	17 (17.0)		
Obesity	691 (33.9)	182 (15.4)	509 (62.6)		430 (56.5)	79 (79.0)		
Body mass index, kg/m^2^ ^b^	28.2 (6.3)	25.4 (4.8)	32.5 (5.9)	<0.001	31.6 (5.6)	36.6 (7.4)		<0.001
Fat mass index, kg/m^2^ ^b^	9.0 (4.0)	7.5 (3.3)	11.2 (4.1)	<0.001	10.9 (3.9)	13.4 (5.1)		<0.001
Waist circumference, cm ^b^	95.5 (15.7)	88.2 (12.3)	106.8 (13.4)	<0.001	104.1 (12.7)	116.4 (15.3)		<0.001
Skeletal muscle mass index, kg/m^2^ ^b^	17.0 (3.3)	15.7 (2.8)	18.9 (3.0)	<0.001	18.4 (2.9)	20.6 (3.3)		<0.001
Appendicular skeletal muscle mass index, kg/m^2^ ^b^	7.9 (1.7)	7.4 (1.5)	8.8 (1.6)	<0.001	8.6 (1.5)	9.5 (1.7)		<0.001
Trunk skeletal muscle mass index, kg/m^2^ ^b^	9.0 (1.7)	8.3 (1.3)	10.1 (1.6)	<0.001	9.8 (1.5)	11.2 (1.7)		<0.001
Liver stiffness measurements, kPa ^b^	5.3 (4.0)	4.7 (2.2)	6.2 (5.7)	<0.001	5.1 (1.2)	14.9 (13.2)		<0.001
Controlled attenuation parameter score, dB/m ^b^	251.9 (61.3)	212.1 (35.7)	313.6 (36.7)	<0.001	309.8 (34.5)	340.4 (38.2)		<0.001
Hypertension ^a^	1416 (74.1)	225 (16.4)	368 (40.5)	<0.001	310 (40.7)	58 (58.0)		0.002
Diabetes ^a^	1925 (95.7)	25 (1.2)	115 (9.2)	<0.001	84 (11.0)	31 (31.0)		<0.001
Insulin resistance ^a c^	543 (60.7)	148 (22.8)	287 (67.0)	<0.001	251 (69.7)	36 (87.8)		0.024
Total Cholesterol, mg/dL ^b^	187.0 (37.7)	181.8 (36.1)	195.0 (38.8)	<0.001	195.9 (39.5)	192.7 (37.5)		0.452
LDL Cholesterol, mg/dL ^b^	110.4 (31.5)	106.9 (30.2)	116.5 (32.7)	0.002	117.4 (31.8)	123.6 (34.2)		0.243
HDL Cholesterol, mg/dL ^b^	53.3 (14.8)	57.3 (14.7)	47.0 (12.5)	<0.001	47.0 (12.6)	43.9 (11.1)		0.017
Triglyceride, mg/dL ^b^	108.1 (88.8)	83.2 (49.7)	150.1 (119.3)	<0.001	145.8 (112.2)	147.5 (73.4)		0.925
Alanine Aminotransferase, IU/L ^b^	23.8 (18.6)	20.4 (17.4)	29.1 (19.3)	<0.001	27.9 (17.3)	41.6 (36.1)		<0.001
Aspartate Aminotransferase, IU/L ^b^	22.5 (13.9)	21.9 (15.2)	23.4 (11.7)	0.141	22.6 (10.4)	30.2 (21.3)		<0.001
C-Reactive Protein, mg/L ^b^	3.3 (6.3)	2.8 (6.7)	4.0 (5.6)	0.003	4.1 (5.7)	5.7 (9.4)		0.022
Fasting Glucose, mg/dL ^b^	104.8 (28.0)	99.2 (16.1)	114.3 (39.0)	<0.001	115.4 (38.9)	147.1 (69.5)		<0.001
Insulin, uU/mL ^b^	11.5 (11.1)	8.1 (6.7)	17.4 (14.2)	<0.001	17.8 (15.1)	23.9 (16.7)		0.016
Glycohemoglobin, % ^b^	5.5 (0.8)	5.3 (0.5)	5.7 (1.0)	<0.001	5.8 (1.1)	6.6 (1.8)		<0.001

MAFLD, metabolic dysfunction-associated fatty liver disease.

a presented as No. (weighted %).

b presented as weighted mean ± SE.

c Insulin resistance was defined as homeostasis model assessment of insulin resistance ≥ 2.5.

Insulin resistance, triglyceride, and fasting glucose were analyzed from fasting subjects only (n = 978).

### Nonlinear associations between muscle mass and CAP, LSM, MAFLD, and significant fibrosis

**Figure 2:**
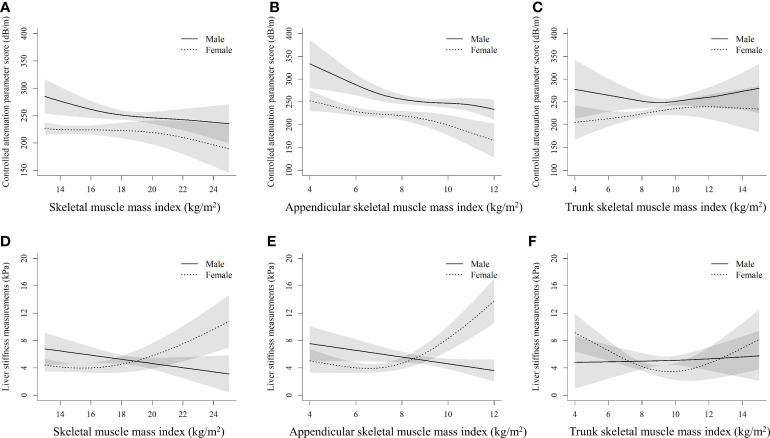
Nonlinear associations of muscle mass index with controlled attenuation parameter score and liver stiffness measurements by sexes **(A)**, **(B)**, and **(C)** stand for the whole-body skeletal muscle mass index, appendicular skeletal muscle mass index, and trunk skeletal muscle mass index with controlled attenuation parameter score, respectively. **(D)**, **(E)**, and **(F)** stand for the whole-body skeletal muscle mass index, appendicular skeletal muscle mass index, and trunk skeletal muscle mass index with liver stiffness measurements, respectively. The models are adjusted for age, race, education, poverty, married status, smoking, alcohol use, health eating index, physical activity, BMI, hypertension, waist circumference, total cholesterol, alanine aminotransferase, aspartate transaminase, and fat mass index.

**Figure 2** shows the sex-specific nonlinear associations between (the whole-body, appendicular, and trunk) SMI and CAP, as well as LSM after multivariate adjustment. There was no evidence of nonlinearity between the whole-body SMI and CAP score (*P_nonlinearity_* = 0.146) in females, but a slightly curvilinear relationship in males (*P_nonlinearity_* = 0.037) (**Figure 2A**). The CAP score was curvilinearly decreased with the increment of appendicular SMI in both sexes (*P_nonlinearity_* < 0.05) (**Figure 2B**). However, a slightly U-shaped relationship (*P_nonlinearity_* = 0.068) between trunk SMI and CAP score was observed in males only, but not in females (**Figure 2C**). Interestingly, both the whole-body and appendicular SMI showed a J-shaped relationship with LSM in females (*P_nonlinearity_* < 0.001), whereas approximately negatively linear associations between them were found in males (**Figures 2D, E**).

**Figure 3:**
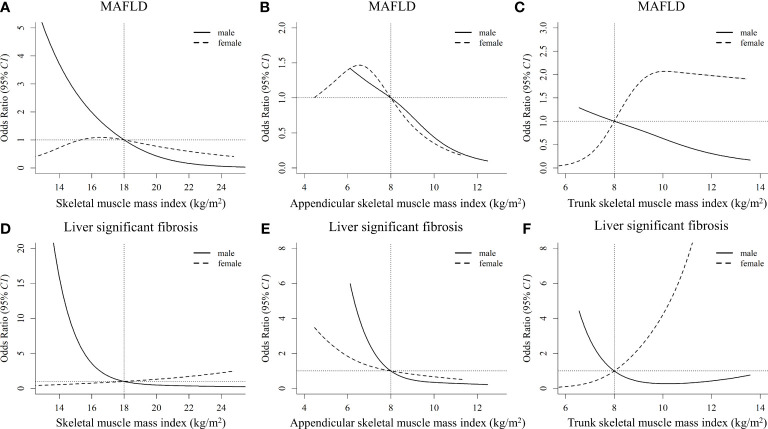
Nonlinear associations of muscle mass index with MAFLD and liver significant fibrosis by sexes **(A)**, **(B)**, and **(C)** stand for the whole-body skeletal muscle mass index, appendicular skeletal muscle mass index, and trunk skeletal muscle mass index with MAFLD, respectively. **(D)**, **(E)**, and **(F)** stand for the whole-body skeletal muscle mass index, appendicular skeletal muscle mass index, and trunk skeletal muscle mass index with liver significant fibrosis, respectively. The models are adjusted for age, race, education, poverty, married status, smoking, alcohol use, health eating index, physical activity, BMI, hypertension, waist circumference, total cholesterol, alanine aminotransferase, aspartate transaminase, and fat mass index. MAFLD, metabolic dysfunction-associated fatty liver disease.

The nonlinear dose-response associations of muscle mass with MAFLD and significant fibrosis derived from restricted cubic spline models are shown in **Figure 3**. Nonlinear relationships between them were observed for all the analyses after multivariable adjustment (all *P_nonlinearity_* < 0.001). Increased whole-body SMI, appendicular SMI, and trunk SMI were associated with reduced risk of MAFLD and significant fibrosis in males, and L-shaped associations between them were observed. However, differing from that in males, the risk of MAFLD increased with the increment of trunk SMI in females, leveling off up to 10-14 kg/m2 (**Figure 3C**). In addition, unlike that in the males, a J-shaped relationship between trunk SMI and the risk of significant fibrosis was observed in females (**Figure 3F**).

### Associations of adequate muscle mass status with MAFLD

**Table 2** categorizes SMI into 2 groups (adequate status defined as SMI in the highest quartile vs non-adequate status) to assess the effect of adequate SMI status on MAFLD. After multivariable adjustment, adequate whole-body SMI and trunk SMI status were not significantly associated with MAFLD in the overall and sex-stratified analyses. A marginal significance was observed between adequate appendicular SMI and reduced risk of MAFLD (OR[95%CI]: 0.61 [0.36~1.03], *P* = 0.083). It is noteworthy that significant sex-specific associations between adequate appendicular SMI and MAFLD have been found (*P_interaction_* = 0.037): compared to those with appendicular SMI below the highest quartile, males and females with adequate appendicular SMI status had 54% (OR[95%CI]: 0.46 [0.25~0.84], *P* = 0.023) and 68% (OR[95%CI]: 0.32 [0.13~0.82], *P* = 0.031) lower risk for MAFLD, respectively. When the sensitivity analysis was performed by using the 274 dB/m CAP scores for steatosis definition, a significantly reduced risk of MAFLD was still observed in the adequate appendicular SMI group (**Table 3**).

**Table 2:** Associations between adequate muscle mass status and MAFLD by sexes.

	All		Male		Female		*P*_interaction_
	*OR* (95%*CI*)	*P* value	*OR* (95%*CI*)	*P* value	*OR* (95%*CI*)	*P* value	
Adequate SMI							0.189
No	1.00 (Reference)		1.00 (Reference)		1.00 (Reference)		
Yes	0.81 (0.44~1.50)	0.517	0.70 (0.30~1.60)	0.411	0.60 (0.27~1.33)	0.227	
Adequate appendicular SMI							0.037^*^
No	1.00 (Reference)		1.00 (Reference)		1.00 (Reference)		
Yes	0.61 (0.36~1.03)	0.083	0.46 (0.25~0.84)	0.023^*^	0.32 (0.13~0.82)	0.031^*^	
Adequate trunk SMI							0.239
No	1.00 (Reference)		1.00 (Reference)		1.00 (Reference)		
Yes	0.85 (0.5~1.43)	0.544	0.89 (0.41~1.93)	0.773	0.53 (0.24~1.16)	0.133	

MAFLD, metabolic dysfunction-associated fatty liver disease; SMI, skeletal muscle mass index; OR, odds ratio; CI, confidence interval. P_interaction_ represents the significance of product term of adequate muscle mass status and sex in the logistic models. The ORs were adjusted for sex (except for sex-stratified analysis), age, race, education, poverty, married status, smoking, alcohol use, HEI, physical activity, BMI, hypertension, waist circumference, total cholesterol, alanine aminotransferase, aspartate transaminase, and fat mass index. * < 0.05.

**Table 3:** Sensitivity analysis: Associations between adequate muscle mass status and MAFLD ^a^ by sexes.

	All		Male		Female		*P*_interaction_
	*OR* (95%*CI*)	*P* value	*OR* (95%*CI*)	*P* value	*OR* (95%*CI*)	*P* value	
Adequate SMI							0.043^*^
No	1.00 (Reference)		1.00 (Reference)		1.00 (Reference)		
Yes	0.98 (0.55~1.75)	0.949	0.91 (0.44~1.89)	0.801	0.63 (0.28~1.42)	0.283	
Adequate appendicular SMI							0.005^*^
No	1.00 (Reference)		1.00 (Reference)		1.00 (Reference)		
Yes	0.66 (0.39~1.10)	0.133	0.52 (0.29~0.95)	0.049^*^	0.29 (0.11~0.75)	0.022^*^	
Adequate trunk SMI							0.375
No	1.00 (Reference)		1.00 (Reference)		1.00 (Reference)		
Yes	1.08 (0.70~1.65)	0.738	0.90 (0.41~1.96)	0.785	1.00 (0.51~1.96)	0.991	

a The hepatic steatosis of MALFD was diagnosed as CAP scores ≥ 274 dB/m.

MAFLD, metabolic dysfunction-associated fatty liver disease; SMI, skeletal muscle mass index; OR, odds ratio; CI, confidence interval. P_interaction_ represents the significance of product term of adequate muscle mass status and sex in the logistic models. The ORs were adjusted for sex (except for sex-stratified analysis), age, race, education, poverty, married status, smoking, alcohol use, HEI, physical activity, BMI, hypertension, waist circumference, total cholesterol, alanine aminotransferase, aspartate transaminase, and fat mass index. * < 0.05.

### Associations of adequate muscle mass status with significant fibrosis

The associations between adequate SMI status and the risk of significant fibrosis are shown in **Table 4**. Significant interactions between adequate whole-body (*P_interaction_* = 0.029) and trunk (*P_interaction_* = 0.008) SMI and sex on significant fibrosis were found in the sex-stratified analyses. Adequate whole-body SMI (OR[95%CI]: 8.68 [1.56~48.12], *P* = 0.025) and trunk SMI status (OR[95%CI]: 7.82 [1.86~32.77], *P* = 0.013) were associated with an increased risk of significant fibrosis in females, but not in males. In addition, individuals with adequate appendicular SMI had a lower risk for significant fibrosis compared to those with appendicular SMI below the highest quartile, of borderline statistical significance (*P* = 0.091).

**Table 4:** Associations between adequate muscle mass status and significant fibrosis by sexes.

	All		Male		Female		*P*_interaction_
	*OR* (95%*CI*)	*P* value	*OR* (95%*CI*)	*P* value	*OR* (95%*CI*)	*P* value	
Adequate SMI							
No	1.00 (Reference)		1.00 (Reference)		1.00 (Reference)		0.029^*^
Yes	1.22 (0.61~2.47)	0.582	1.07 (0.41~2.76)	0.896	8.68 (1.56~48.12)	0.025^*^	
Adequate appendicular SMI							
No	1.00 (Reference)		1.00 (Reference)		1.00 (Reference)		0.164
Yes	0.39 (0.14~1.08)	0.091	0.59 (0.21~1.61)	0.317	1.67 (0.38~7.26)	0.506	
Adequate trunk SMI							
No	1.00 (Reference)		1.00 (Reference)		1.00 (Reference)		0.008^*^
Yes	1.48 (0.71~3.09)	0.307	1.15 (0.54~2.45)	0.715	7.82 (1.86~32.77)	0.013^*^	

SMI, skeletal muscle mass index; OR, odds ratio; CI, confidence interval. P_interaction_ represents the significance of product term of adequate muscle mass status and sex in the logistic models. The ORs were adjusted for sex (except for sex-stratified analysis), age, race, education, poverty, married status, smoking, alcohol use, HEI, physical activity, BMI, hypertension, waist circumference, total cholesterol, alanine aminotransferase, aspartate transaminase, and fat mass index. * < 0.05.

### Most influential predictors of MAFLD and significant fibrosis

**Figure 4:**
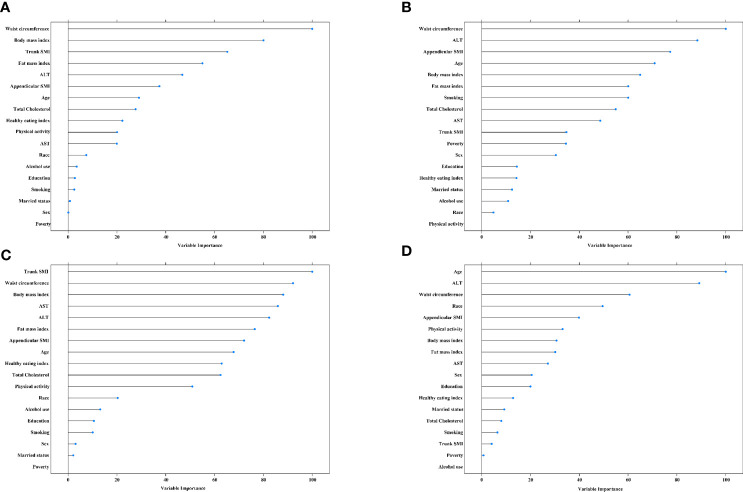
Variable importance for predictors of MAFLD and significant fibrosis **(A)**, random forest for MAFLD. **(B)**, logistic regression model for MAFLD. **(C)**, random forest for significant fibrosis. **(D)**, logistic regression model for significant fibrosis. The predicators included age, race, education, poverty, married status, smoking, alcohol use, health eating index, physical activity, BMI, hypertension, waist circumference, total cholesterol, alanine aminotransferase, and fat mass index. MAFLD, metabolic dysfunction-associated fatty liver disease. SMI, skeletal muscle mass index. ALT, alanine aminotransferase. AST, aspartate transaminase.

**Figure 5:**
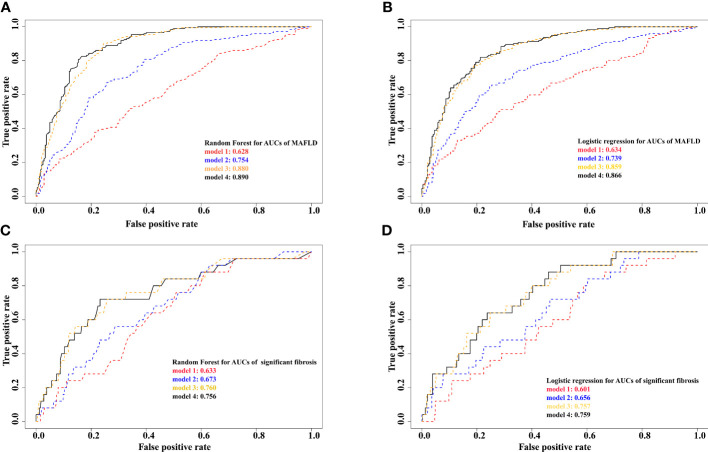
Prediction results from different models for MAFLD and significant fibrosis **(A)**, random forest for MAFLD. **(B)**, logistic regression for MAFLD. **(C)**, random forest for significant fibrosis. **(D)**, logistic regression for significant fibrosis. Model 1 adjusted for age, race, education, poverty, married status, smoking, alcohol use, HEI, physical activity, hypertension. Model 2 further adjusted for total cholesterol, alanine aminotransferase, and aspartate transaminase. Model 3 further adjusted for BMI, waist circumference, and fat mass index. Model 4 further adjusted for appendicular and trunk skeletal muscle mass index. MAFLD, metabolic dysfunction-associated fatty liver disease.

The top three contributors to MAFLD based on variable importance were not the same between prediction methods: waist circumference ranked first in both methods whereas trunk SMI and appendicular SMI ranked third in random forest and logistic regression models, respectively (**Figures 4A, B**). For significant fibrosis, Trunk SMI ranked the first in the random forest model (**Figure 4C**), and appendicular SMI ranked fifth in the logistic regression model (**Figure 4D**). The performance in predicting MAFLD and significant fibrosis was compared among models with different adjusted covariates (**Figure 5**). With the fully adjusted model that further took appendicular and trunk SMI into consideration, the AUCs for MAFLD were 0.890 and 0.866 in random forest and logistic regression models, respectively. Regarding significant fibrosis, AUCs showed minor changes when further adjusting skeletal muscle.

## Discussion

Using a nationally representative US population sample, we observed that adequate appendicular skeletal muscle mass was associated with a lower risk for MAFLD in both sexes but with a significantly different scale of the associations between sexes. Interestingly, significantly sex-specific associations between adequate trunk skeletal muscle mass and significant fibrosis were found in the current study: adequate trunk skeletal muscle mass was related to the increased risk of significant fibrosis in females, but not in males.

Skeletal muscle, which accounts for the majority of post-prandial glucose uptake, is considered to be related to obesity-related metabolic abnormalities (19). The diagnosis of new terminology MAFLD is based on the recognition of underlying abnormalities in metabolic health (1), and thus skeletal muscle is supposed to be a potential player in MAFLD. Emerging studies have explored the relationships between skeletal muscle mass and NAFLD, hepatic steatosis, as well as significant liver fibrosis (8, 9, 20–23). However, most of them used less accurate surrogate measures, such as handgrip strength (8, 9, 22) and fatty liver index (23). Leveraging data from the Korean National Health and Nutrition Examination Surveys (KNHANES), Lee Sung-Bum found that handgrip strength was inversely associated with HSI, and the prevalence of NAFLD defined by HSI decreased in the quartile groups of handgrip strength (9). Being partially consistent with our results, they also observed that the risk for NAFLD in females (OR[95%CI]: 0.30 [0.22–0.40]) was more relevant to muscle strength than that in males (OR[95%CI]: 0.42 [0.32–0.55]), though without testing the difference by a statistical method. A cross-sectional analysis of 613 Chinese middle-aged individuals with biopsy-proven NAFLD showed that lower skeletal muscle mass combined with abdominal obesity was strongly associated with the presence of non-alcoholic steatohepatitis only in males (24). However, another cross-sectional study performed in Chinese patients with type 2 diabetes indicated that the appendicular skeletal muscle mass to visceral fat area ratio was inversely associated with the risk of NAFLD only in females (25).

Recent evidence from a prospective cohort study with a large sample from the UK Biobank investigated the relationships between skeletal muscle mass estimated by bioimpedance and NAFLD (8). Consistently, they found a lower risk of NAFLD was associated with the increment of skeletal muscle mass, but the associations between skeletal muscle mass distribution and NAFLD were not further analyzed. Our research took further steps in this regard. With more accurate skeletal muscle mass data assessed by DXA, instead of the whole-body and trunk skeletal muscle mass, we found that a lower risk of MAFLD was only related to the appendicular skeletal muscle mass. Another KNHANES study found an unstable association between low skeletal muscle strength and significant fibrosis after multivariable adjustment (22). Whereas in our current study, the higher risk for significant fibrosis in females with adequate trunk skeletal muscle mass remained after adjusting for potential confounders. Inconsistently, a cross-sectional analysis of 487 patients with T2DM indicated that low skeletal muscle mass is independently associated with liver fibrosis (26).

A decline in muscle mass, or sarcopenia, is commonly observed among patients with liver disease, with a prevalence ranging from 20% to 70% (27). The biological mechanisms underpinning the associations between skeletal muscle mass and chronic liver diseases are related to insulin resistance, oxidative stress, chronic inflammation, insufficient physical activity, myokines, and hepatokines (28). As an endocrine organ, skeletal muscle can secrete a panel of cytokines named “myokines”, which exert a paracrine regulatory function on distant organs (i.e., adipose tissue, bone, and liver) and counteract the condition of chronic low-grade inflammation raised by metabolic disorders (29). Beyond the beneficial effects of skeletal muscle mass on MAFLD, our results also indicated a protective role of the appendicular skeletal muscle mass rather than trunk skeletal muscle mass that played. By using NHANES III survey data, Peng TC also found severe hepatic steatosis was associated with a lower risk of sarcopenia when using the height-adjusted SMI (30). Even though it is still not well understood why the associations vary between regional skeletal muscle mass distribution MAFLD and significant fibrosis, it is reasonable to hypothesize that the lipid-rich environment in which the trunk skeletal muscles are embedded, surrounded by both visceral and subcutaneous adipose tissue, could influence their intra-muscular fat content (31). There is consistent evidence that ectopic adipose tissue infiltration into skeletal muscle (i.e., myosteatosis) plays an important role in the risk of metabolic disorders (32). Hsieh YC et al. found severe myosteatosis was significantly associated with fibrosis progression rather than sarcopenia (33). In a prospective study consisting of 52 obese patients, myosteatosis was the strongest factor associated with fibrosis (34). Previous studies demonstrated that myosteatosis is positively associated with insulin resistance and systemic inflammation, and therefore linked with early steatohepatitis (35–37). The evidence offered a plausible explanation for the adverse effect of trunk skeletal muscle mass on significant fibrosis observed in the present study. Additionally, our result showed sex-related differences in the associations between skeletal muscle mass and MAFLD and significant fibrosis. A recent study performed in the same population reported sex-specific differences in the role of fat distribution on NAFLD and liver fibrosis (38). Similarly, their results showed that the effect of android fat deposition on fibrosis was only evident in females. The sex differences in hormones, muscle capillary density, muscle fiber type composition, and expressed estrogen receptors, which may affect glucose and fatty acid oxidative and storage capacities in muscle, could lead to these sex-specific associations observed in the present study (39).

The utilization of DXA and transient elastography in the present study provides more accurate data on skeletal muscle mass and MAFLD diagnosis than surrogate measures, such as handgrip strength and fatty liver predictor. However, there are several limitations in our study worth noting. First, the trunk composition measured by DXA involves substantial prediction and thus is less accurate than the composition in the limbs (40). Additionally, DXA cannot measure muscle lipid content and the location of fat storage within or surrounding myocytes. Thus, the causality between skeletal muscle and MAFLD needs to be further investigated using more accurate measurements, such as computed tomography and magnetic resonance imaging. Second, the temporal causality of the observed associations can not be inferred due to the nature of the cross-sectional design. Therefore, prospective studies are needed to assess the impact of skeletal muscle mass on MAFLD to validate our results. Third, the unmeasured residual confounders may limit the validity of our results. Fourth, the accuracy of the diagnosis of liver steatosis and fibrosis by transient elastography might be affected by body fat.

To the best of our knowledge, this is the first investigation of regional and sex-specific associations between skeletal muscle mass and MAFLD and significant fibrosis using a representative sample of the US general population. In our research, the appendicular skeletal muscle mass was suggested to have beneficial effects on MAFLD in both sexes, whereas the trunk skeletal muscle mass may increase the risk of significant fibrosis in females. These findings indicated that future studies on the effects of skeletal muscle on MAFLD should account for regional distribution and sex-specific differences. Given that MAFLD is the most common chronic liver disease and its inverse associations observed in the present study, improving appendicular skeletal muscle mass among the general population might be helpful to prevent this increasing public health concern.

## Data availability statement

Publicly available datasets were analyzed in this study. This data can be found here: https://www.cdc.gov/nchs/nhanes/index.htm.

## Ethics statement

The studies involving human participants were reviewed and approved by the Centers for Disease Control and Prevention Institutional Review Board. The patients/participants provided their written informed consent to participate in this study.

## Author contributions

Conceptualization and methodology, PX and PL. Formal analysis, PX. Writing—original draft preparation, PL. Writing—review and editing, PX, PG, and JW. Supervision, PL. All authors have read and agreed to the published version of the manuscript.

## Funding

PX was supported by Beijing Natural Science Foundation (7214277). PL was supported by funding from Scientific Research Common Program of Beijing Municipal Commission of Education (KM202110025003).

## Acknowledgments

We gratefully thank all participants in the study and thank all the staff of the NHANES.

## Conflict of interest

The authors declare that the research was conducted in the absence of any commercial or financial relationships that could be construed as a potential conflict of interest.

## Publisher’s note

All claims expressed in this article are solely those of the authors and do not necessarily represent those of their affiliated organizations, or those of the publisher, the editors and the reviewers. Any product that may be evaluated in this article, or claim that may be made by its manufacturer, is not guaranteed or endorsed by the publisher.
